# Virus Nanoparticles Mediated Osteogenic Differentiation of Bone Derived Mesenchymal Stem Cells

**DOI:** 10.1002/advs.201500026

**Published:** 2015-06-25

**Authors:** Kamolrat Metavarayuth, Pongkwan Sitasuwan, Jittima Amie Luckanagul, Sheng Feng, Qian Wang

**Affiliations:** ^1^ Department of Chemistry and BiochemistryUniversity of South Carolina631 Sumter StreetColumbiaSC29208USA; ^2^ Department of Food and Pharmaceutical ChemistryFaculty of Pharmaceutical SciencesChulalongkorn University254 Phayathai Rd., WangmaiPathumwanBangkok10330Thailand

**Keywords:** biomaterials, mesenchymal stem cells, nanotopography, osteogenesis, virus nanoparticles

## Abstract

There are few methodologies that allow manipulating a biomaterial surface at nanometer scale, which controllably influence different cellular functions. In this study, virus nanoparticles with different structural features are selected to prepare 2D substrates with defined nanoscale topographies and the cellular responses are investigated. It is demonstrated that the viral nanoparticle based substrates could accelerate and enhance osteogenesis of bone derived mesenchymal stem cells as indicated by the upregulation of osteogenic markers, including bone morphogenetic protein‐2, osteocalcin, and osteopontin, at both gene and protein expression levels. Moreover, alkaline phosphatase activity and calcium mineralization, both indicators for a ­successful bone formation, are also increased in cells grown on these nanoscale possessed substrates. These discoveries and developments present a new paradigm for nanoscale engineering of a biomaterial surface.

## 1 Introduction

It is well established that cell–material interactions regulate numerous cellular functions.^1^ Biological processes such as adhesion, growth, differentiation, and apoptosis, are controlled by cell shape and cytoskeletal organization which is directed by cell–surface interactions.^2^ Meanwhile, the surface chemistry and topography of materials play a very crucial role in altering cell behaviors at many stages of cell growth and development.[[qv: 1c]],^3^ Although the dimensions of mammalian cells are in the order of a few micrometers, cellular sensing of the external environment and interaction with biomaterials occurs at the nanometer level.^4^ Cell interactions with nanometric surfaces often result in a specific sequence of gene and protein regulations. These series of events initiate as early as the cell begins to sense the surrounding environment. Therefore, the understanding of various topographical cues that are responsible for cellular behaviors is a key to advance tissue engineering.

In general, topographical cues can be classified as: 1) the roughness of the underlying surface, 2) the ligand‐display pattern and density, 3) the size and shape of the contact area for cell spreading, and 4) the geometry of topological features at a nanometer scale.^5^ Reviewing the effect of individual cues is often complicated due to the difficulty in controlling and altering particular topographical features while preserving others. Micro/nanofabrication techniques are required to enable the recapitulation of topographical cues in the cell niche in a controllable and reproducible fashion. Examples of these technologies are mechanical roughening,^6^ nano‐ and microindentation, and substrate‐templating using a well‐defined relief to impart topography with solvent‐casting, electrodeposition, chemical‐vapor depositions, or compression‐molding processes.^7^ These engineered micro/nanoscale topographical cues mimic the micro/nanoscale features in the physiological environment, which can be used to demonstrate how individual cues or the combination of topographical cues affect a particular cellular response. However, all these methods suffer from the laborious process, the inability of predictably generating chemistry and topography in a simultaneous fashion, the requirement for high‐cost equipment, and the limited class of material can be used.[[qv: 2c]],^8^

Certain virus particles, especially the plant viruses, have well characterized 3D structure and can be produced in high yield and purity.^9^ The multivalent organization of the coat proteins make the viral particles powerful scaffolds for display of a variety of functional groups via chemical conjugation or genetic modification.[[qv: 9a]],^10^ In the past two decades, using virus particles as building blocks, novel materials with unique structural features have been developed for a wide range of applications, including electronics, sensing, gene/drug delivery, bioimaging, and vaccine development.[[qv: 10c]],^11^ However, so far there is no systematic investigation about how the nanoscale topographical cues of various plant virus particles coated substrates impact cell behaviors, specifically, osteogenesis of bone derived mesenchymal stem cells (BMSCs).

**Figure 1:**
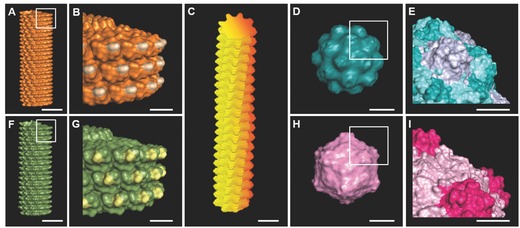
A–I) Molecular models shows surface topography of plant viruses used in this study. A,B) Tobacco mosaic virus (TMV); C) potato virus X (PVX); D,E) turnip yellow mosaic virus (TYMV); F,G) turnip vein clearing virus (TVCV); H,I) cowpea mosaic virus (CPMV). Scale bar indicates 10 nm in (A), (C), (D), (F), and (H) and 5 nm in (B), (E), (G), and (I). The models were generated using Pymol (www.pymol.org) with coordinates obtained from RCSB protein data bank.

From our unexpected, yet significant, observation that rodlike plant virus nanoparticle, tobacco mosaic virus (TMV) coated two dimensional (2D) substrate dramatically accelerates osteogenesis of BMSCs. The study suggested that the virus does not act as soluble inducer as supplementing cell culture media with TMV solution failed to mediate the differentiation.^12^ We have hypothesized that shape of virus nanoparticle and/or nanoscale topography provided by surface structure of virus particle is necessary for the enhanced osteogenesis. Therefore, in this study, we generate a series of plant virus nanoparticles coated substrates with distinct morphology and nano­topography via electrostatic interaction. We applied these virus based scaffolds to investigate cellular responses to two types of the topographical cues: 1) the geometry of topological features by testing effects of three different viral particle shapes including rigid rod, spherical, and flexible fiber; and 2) the size and shape of the contact area for cell spreading at nanoscale level by utilizing viral particles with same shape but different in nanoscale features, constructed from different structure of coat protein that assembles around virus genomic material as shown in **Figure** **1**. Our results show that some of these virus based scaffolds accelerate and enhance osteogenic differentiation of BMSCs. This finding presented here may provide a new route for enhancing the performance of orthopedic implants by regulating stem cell differentiation with nanotopography.

## 2 Results and Discussion

### 2.1 Fabrication of Virus‐Coated Scaffolds by Layer‐by‐Layer Deposition Method

We fabricated 2D virus based substrates from five plant viruses which can be categorized into three groups by morphology of the viral particles rod shape virus; TMV and turnip vein clearing virus (TVCV), filamentous virus; potato virus X (PVX), and spherical virus; turnip yellow mosaic virus (TYMV) and cowpea mosaic virus (CPMV). Not only are these plant viruses morphologically different, but also they are nanotopographically dissimilar as shown in Figure 1.

**Figure 2:**
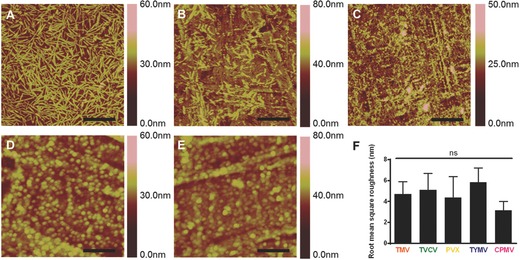
Representative AFM images showing the coverage of PDL coated substrate with different virus nanoparticles indicate the viral particles, A) TMV; B) TVCV; C) PVX; D) TYMV; and E) CPMV, are mostly intact and fully cover the coating area. F) Root mean square roughness of different virus nanoparticles coated substrates by AFM analysis. Scale bars indicate 1.25 μm in (A)–(C) and 0.5 μm in (D) and (E). The data are expressed as mean ± s.d. (*n* = 4) ns indicates nonsignificant and *p* > 0.05 based on ANOVA.

Since all these viral particles have isoelectric pH less than 5.5, overall surface charges on these particles are negative in neutral condition. Via an electrostatic interaction, negatively charged viral particles can be strongly adsorbed on 12‐well plates coated with poly‐*d*‐lysine (PDL), a positively charged biocompatible polymer. And the interaction helps to retain the viral particles on surface of the substrates. By depositing structurally and nanotopographically distinct viral particles on PDL coated substrate, we can readily construct an array of virus‐coated scaffolds with various topographies offered by the intrinsic morphology and micro/nanotopography of each viral particle. The presence of viral particles on PDL coated surface was confirmed by atomic force microscopy (AFM) (**Figure** **2**). The AFM images also show a nearly complete coverage of substrates by intact viral particles. The virus particles are randomly adsorbed on 12‐well plates coated with poly‐*d*‐lysine, however, some area of the virus coated substrates appeared to show direction of virus particles coating under AFM. This coating pattern results from the natural irregularity of the cell culture surface of 12‐well plate. To prevent the effect of plate pattern, same lot of 12‐well plates was used throughout this study. The virus substrates have been characterized in term of root mean square roughness from data collected from AFM images (*n* = 4). There is no significant difference of microscale roughness across the virus coated substrates, created from deposition of numerous virus particles on the substrate surface, across these five virus substrates. However, different type of virus particle has dissimilar nanoscale topography provided by surface of each particle of virus as shown in Figure 1.

### 2.2 Viral Particles Coated Substrates Promote Osteogenesis

**Figure 3:**
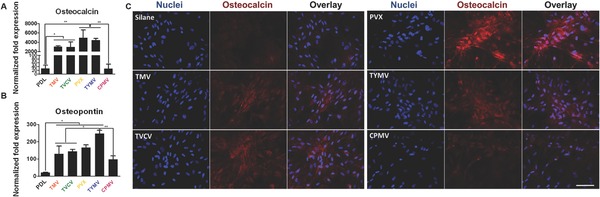
The expression of osteogenic markers in BMSCs cultured on PDL and different virus nanoparticles coated substrates under osteogenic conditions. Quantitative real‐time PCR analysis (RT‐qPCR) showed upregulation of A) osteocalcin and B) osteopontin in cells grown on TMV, TVCV, PVX, and TYMV (but not on CPMV) coated substrates at 7 d after osteogenic induction. C) Immunohistochemical staining reveals that osteocalcin, a canonical osteogenic marker, is exclusively located in cell aggregates growing on TMV, TVCV, PVX, and TYMV substrates (not for CPMV coated substrate). Color representation: nucleus (blue), osteocalcin (red). Scale bar is 100 μm. The data were expressed as mean ± s.d. (*n* = 3, *p ≤ 0.05, **p ≤ 0.01 based on ANOVA).

To investigate the effect of surface topography on osteogenesis, we culture BMSCs on PDL coated substrate and the five virus‐based substrates and study the osteoblastic differentiation. BMSCs are isolated and cultured as reported in literature. The purity of the stem cells populations has been previously verified with several stem cells markers such as Cluster of Differentiation 73 and 90 (CD73 and CD90).^13^ The difference in the expression of bone morphogenetic protein‐2 (BMP2) gene, an early osteogenic marker,[[qv: 12b]] among BMSCs cultured on PDL and virus substrates were recorded at 6 h after osteoinduction (Figure S1, Supporting Information). Moreover, after 7 d of induction, osteocalcin (BGLAP) and osteopontin (SPP1) genes expressions were higher compared to uninduced BMSCs (**Figure** **3**). These two genes are noncollagen genes actively involved during proliferation period. Osteocalcin is a specific marker for the osteoblast differentiation and mineralization, and is expressed exclusively during the postproliferative period and reaches its maximum expression during mineralization and accumulates in the mineralized bone.^14^ Osteopontin is known to serve as a bridge between the cells and the hydroxyapatite through the arginine‐glycine‐aspartic acid (RGD) peptide and polyaspartate sequences present in it. It is one of the early markers of osteoblastic differentiation.^15^ We observed significant changes in the expression of all three osteospecific genes in cells plated on the virus based substrates, except CPMV coated substrate, compared to cells grown on bare PDL substrate. Interestingly, in the case of spherical‐shaped viral particles, while TYMV coated substrates increased BMP2 gene expression by fourfold and dramatic increment of BGLAP and SPP1 were observed, there was no significant difference in these gene expressions between cells plated on PDL and CPMV substrates.

**Figure 4:**
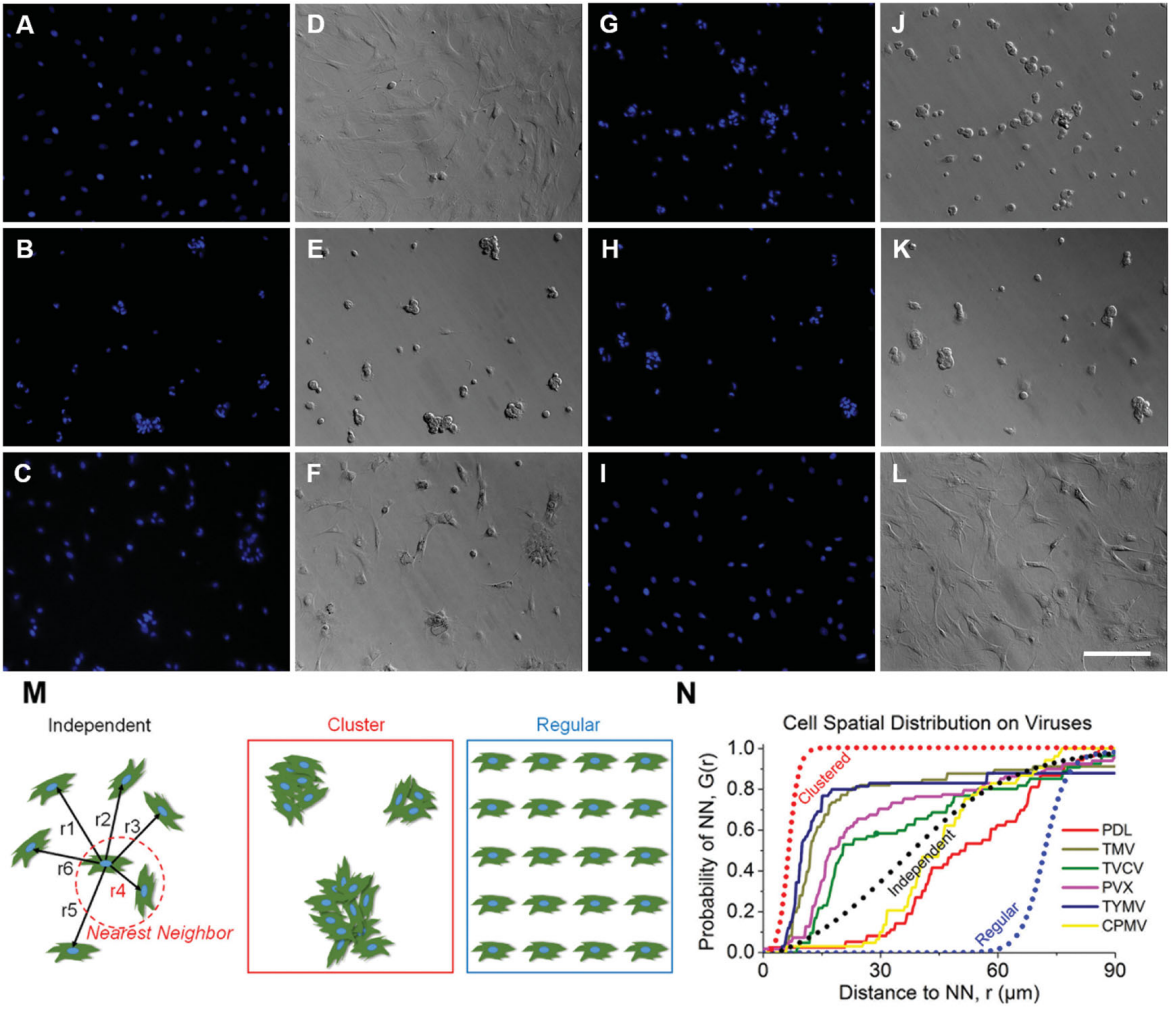
Nearest neighbor analysis of BMSCs cultured on PDL and virus substrates. A–C,G–I) DAPI immunohistochemical staining and D–F,J–L) bright field microscopy images of BMSCs on A,D) PDL, B,E) TMV, C,F) TVCV, G,J) PVX, H,K) TYMV, and I,L) CPMV. M) Schematic diagrams of the nearest neighbor analysis. In this analysis the distribution of cells can range from independent (represented by a theoretical Poisson's distribution), to clustered, or regular. N) Plot of BMSCs spatial distribution on PDL control and virus substrates demonstrated cluster growth pattern which correlated to appearance of cells nodules on TMV, TVCV, PVX, and TYMV virus coated substrates. Scale bar is 200 μm.

In consistence with gene expression data, immunofluorescence imaging of BMP2 (Figure S2, Supporting Information) and osteocalcin (Figure 3C) revealed that the morphogens are localized in the cell aggregates on the four virus coated substrates. BMSCs cultured on TMV, TVCV, PVX, and TYMV develop greater cell nodules, a notable feature of BMSCs undergoing osteogenesis. In order to quantify the differences in the spatial distributions of cells on each substrates, we acquired the coordination of cells and applied nearest neighbor analysis.^16^ The spatial distributions of BMSCs on TMV, TVCV, PVX, and TYMV substrates were similar to the theoretical “cluster” distribution, which indicates cells tend to cluster to form the cell nodules (**Figure** **4**). On the other hand, the spatial distribution of BMSCs on PDL and CPMV were similar to the “independent” distribution and shifted toward “regular” distribution. The data suggest that TMV, TVCV, PVX, and TYMV coated substrates are more favorable to the osteogenesis of BMSCs than PDL and CPMV substrates.

These cell clusters displayed robust positive staining for BMP2 in cell aggregates (Figure S2, Supporting Information). No fluorescence signal was detected in cells grown on PDL control and CPMV substrates. Similarly, immunohistochemical staining of osteocalcin at 14 d indicates that the canonical osteogenic marker was exclusively found in cells aggregates on TMV, TVCV, PVX, and TYMV substrates.

**Figure 5:**
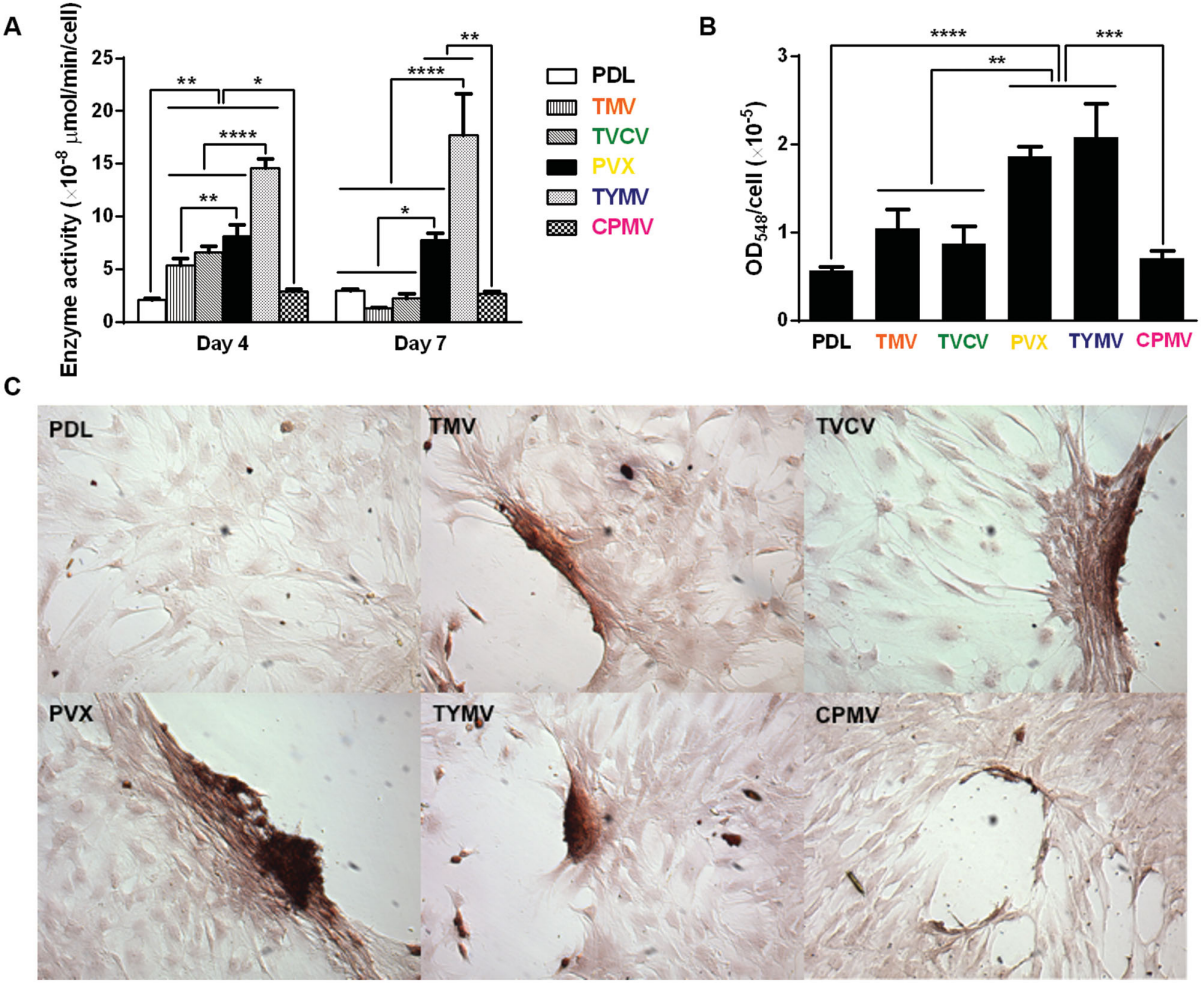
Cytochemical analysis of the bone differentiation process of BMSCs on PDL and viruses coated substrates at 4 and 7 d after osteogenic induction. A) Alkaline phosphatase activity of cells cultured on different substrates. The data are expressed as mean ± s.d. (*n* = 3, *p ≤ 0.05, **p ≤ 0.01, ****p ≤ 0.0001 based on ANOVA). B) Alizarin red staining of each sample at day 7. Cells on virus substrates are positively stained for calcium deposition, whereas negatively stained is observed on PDL substrates. The data are expressed as mean ± s.d. (*n* = 3, **p ≤ 0.01, ***p ≤ 0.001, ****p ≤ 0.0001 based on ANOVA). C) Absorbance at 548 nm normalized to cell number to indicate a relative amount calcium deposit at day 7 stained by alizarin red solution. The mineralization of cells on TMV substrates doubles that of PDL, while PVX and TYMV substrates increase the mineralization by fourfold. TVCV substrates slightly increase the mineralization of cells compare to PDL control substrates. These evidences suggest an improvement in osteogenesis by virus coated substrates.

In addition to the analysis of osteo‐specific markers, alkaline phosphatase (ALP) activity and calcium mineralization supported the osteogenic differentiation of cells on the four virus based scaffolds. ALP is an early marker of osteogenesis and its activity mediates matrix mineralization.^17^ Cytochemical analysis of the osteogenesis process of BMSCs on PDL and virus coated substrates at day 4 and 7 after osteogenic induction suggested that cells on TMV, TVCV, PVX, and TYMV substrates had an increase in ALP activity at day 4, whereas CPMV substrates did not alter the enzyme activity when compared to PDL control. The enzyme activity drops to baseline at day 7 for cells on TMV and TVCV substrates (**Figure** **5**A). It is possible that cells on these two virus substrates undergo differentiation and reach mineralization period earlier than cells on other substrates since alkaline phosphatase activity rises during cell proliferation and achieves maximum level as the culture progresses into mineralization stage. However, cellular level of ALP declines as mineralization progresses.^18^ Additionally, cells on the four virus substrates at day 7 were positively stained by Alizarin red S which showed deep red color for calcium deposition in large cell nodules, whereas negatively stained was observed on PDL substrates (Figure 5C). Cells on CPMV substrate only formed small nodules that were also stained with Alizarin red S. Quantification of dissolved Alizarin red S dye from cells nodules by UV–vis absorbance indicated that the mineralization of cells on TMV substrates doubled that of PDL, and PVX and TYMV substrates increased the mineralization by fourfold, while TVCV substrates slightly increased the mineralization of cells compared to PDL control substrates but not statistically significant (Figure 5B). However, the calcium mineralization is an accumulation process, longer incubation time of cells on these substrates could increase the difference in calcium deposition between each substrate and may increase difference of the mineralization between cells on TVCV and PDL coated substrates. Cells on CPMV substrate have comparable calcium mineralization to cells on PDL control. The combined results from quantitative real‐time PCR analysis (RT‐qPCR), immunohistochemical staining, nearest neighbor analysis, enzyme activity, and calcium mineralization unambiguously indicate that TMV, TVCV, PVX, and TYMV substrates can accelerate and enhance osteogenesis of BMSCs. The accelerated osteogenic differentiation of BMSCs on TMV and TYMV substrates has been demonstrated before in our previous studies when BMSCs were cultured on the viruses coated APTES glass coverslips.^12^, ^19^ In this study, we have confirmed that it is the topography created by deposition of virus nanoparticles on substrates, not underlying material, which mediates such differentiation, as we apply different backup material; poly‐*d‐*lysine coated tissue culture plate. We also expand the library of virus based substrates to include another morphology of virus nanoparticle; flexible fiber (PVX) as well as other types of virus nanoparticles with dissimilar nanoscale topography (TVCV and CPMV).

### 2.3 Nanotopography of Viral Based Scaffolds Alters Cells Morphology and Induces Differentiation

**Figure 6:**
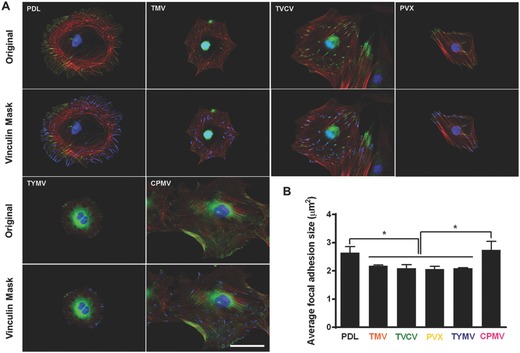
Immunochemical staining showing the difference in vinculin size of cells on PDL or virus coated substrates for 24 h. A) Immunofluorescence images of cells on different substrates at 24 h prior to osteoinduction (top panel). Color representation: nucleus (blue), vinculin (green), and phalloidin (red). The bottom panel demonstrates vinculin masking and selection of vinculins for size analysis. The selected vinculin spots are highlighted in blue. Scale bar is 50 μm. B) Average vinculin size of cells on different substrates. The data were expressed as mean ± s.d. (*n* = 3, * represents *p* ≤ 0.05 based on ANOVA).

The majority of cells cultured on the four virus substrates have noticeably smaller size at 24 h after seeding compared to those on PDL and CPMV substrates. Previous study illustrated that cell shape and size are associated with adhesion strength of cells on a substrate.^20^ Additionally, several reports showed that integrin‐mediated focal adhesion (FA) is an important regulator of osteogenesis.^21^ It is hypothesized that too strong substrate binding may inhibit osteogenic differentiation. Mendonça et al. observed higher osteogenic differentiation of stem cells that attached looser on rough titanium disks than strongly attached cells on smooth substrate.^22^ This could possibly be due to the limitation of cells movement or migration. Strength of cell adhesion and larger focal adhesion size are correlated to an increase in localization of vinculin.^23^ Therefore, we investigated cell adhesion on virus substrates by using fluorescence imaging of vinculin, a protein of focal adhesion complexes (FAC), to analyze average focal adhesion size of cells grown on PDL and virus substrates for 24 h prior to osteoinduction. Vinculin signals were captured by fluorescence microscopy for size analysis by Slidebook 5 software. The data revealed the reduction in vinculin size of cells on TMV, TVCV, PVX, and TYMV but not CPMV substrates (**Figure** **6**).

These results suggest that BMSCs attached to the four virus substrates weakly, whereas larger size of FACs dictates stronger cell–substrate adhesion in PDL and CPMV substrates.^24^ The significantly smaller FA size for cells on the four virus substrates might increase cellular motility and facilitate the formation of cell aggregates within 6 h of osteoinduction. The larger FA size observed in CPMV sample, which did not improve osteogenic differentiation, might be due to the expression of vimentin binding ligand on CPMV coat proteins.^25^ The vimentin cytoskeleton was shown to regulate focal contact size and help stabilize cell–matrix adhesion in endothelial cells.^26^ Since major cytoskeletal component of mesenchymal cells is vimentin, the presence of vimentin binding ligand on CPMV substrate could supply additional adhesion points and consequently leads to higher adhesion strength of cells cultured on CPVM coated substrate, therefore mitigating cell migration and differentiation.

Several reports previously described that elongated shapes and geometries that present features of subcellular concavity at the cell perimeter increase the cytoskeletal tension in mesenchymal stem cells (MSCs), thus promoting the preference for osteogenesis.^27^ These similar geometries of BMSCs were also observed in our study. Representative actin and vinculin immunofluorescent heat maps of cells initially adhere on PDL and each virus coated substrates suggests that cells on TMV, TVCV, PVX, and TYMV were more elongated with higher actin stress fiber on the long axis of cells, and the majority of them had concave features that led to high cytoskeleton tension in the region. Furthermore, vinculin of cells that grew on these four substrates were highly localized at the protrusion area which was different from those of cells on PDL and CPMV coated substrates. The majority of cells on PDL and CPMV coated substrates were round in shape with evenly distributed actin filaments and vinculin around cell perimeter (Figure S3, Supporting Information). Moreover, overall morphology of cells on each virus substrates, which can be investigated from Figure 4 and Figure S2 (Supporting Information), reveals that cells on CPMV have more spread out shape compared to cells on other virus substrates. These data of morphology and immunofluorescence heat maps along with small FA size suggest that loose attachment of cells on unfriendly four virus, TMV, TVCV, PVX, and TYMV, coated substrates result in cytoskeleton tension, thereby enhancing osteogenic differentiation of BMSCs.

Interestingly, data from this study suggest that the effect of nanoparticle morphology on differentiation is negligible. As observed from all experiments, osteogenic differentiation is comparable in cells cultured on substrates coated with different shape of virus.

## 3 Conclusion

A series of assorted micro‐/nanoscale features possessed 2D peptide based scaffolds can be simply constructed from structurally distinct viral bionanoparticles by using fundamental electrostatic interaction. These virus based 2D scaffolds were used to investigate osteogenesis of BMSCs. The combined results from RT‐qPCR and immunostaining of BMP2 suggest an early osteogenesis of cells on TMV, TVCV, PVX, and TYMV coated substrates as early as 6 h after osteoinduction. Furthermore, the confirmation of the strong commitment in osteoinduction in longer term was evidenced by RT‐qPCR and immunostaining of osteocalcin and osteopontin, as well as enzyme activity, and calcium mineralization. These results suggest that topographies created by TMV, TVCV, PVX, and TYMV coated substrates stimulate and enhance osteogenic differentiation. The underlying mechanisms of the observation are proposed that the stress created by the unfavorable surface from the four viral nanoparticles causes the reduction in FA size, which in turn increases cell motility and facilitates the formation of cell aggregates. The unfavorable surface may also obstruct cell spreading therefore increased cytoskeleton tension which results in high aspect ratio or subcellular concavity at the cell perimeter, thus promoting osteogenesis.

Further investigation about topography‐induced differentiation is necessary for a better understanding of how surface topography provided by viral particles affect cell–material adhesion complex and facilitate the differentiation. Additionally, a continued study can be done on the investigation of the alignment or patterning of virus particles on the cellular responses as the unique structure or morphology of virus particles make them feasible for hierarchical structure formation in both 2D and 3D substrates.^28^ More importantly, it will be very interesting to study if our discovery can be extended to other synthetic substrates and employed in clinical tissue engineering applications.

## 4 Experimental Section

*Virus Purification from Infected Leaves*: Purification of TMV, TVCV, TYMV, and CPMV were done by first, infected leaves were blended with three volumes of 0.1 m potassium phosphate buffer pH 7.0 and 0.1% β‐mercaptoethanol. The mixture was filtered, and the filtrate was subjected to centrifugation to remove bulk plant material. The supernatant was collected and clarified by adding an equal volume of CHCl_3_/1‐butanol (v/v = 1:1). The aqueous layer was then collected followed by precipitation of virus with 4% PEG 8K and 0.2 m NaCl. The pellet was centrifuged and resuspended in buffer before it was subjected to low speed centrifuge to remove PEG. The virus in supernatant was finally pelleted by ultracentrifugation and resuspended in buffer. For purification of PVX, infected leaves were blended with two volumes of 0.1 m potassium phosphate buffer pH 8.0, 10% ethanol, and 0.1% β‐mercaptoethanol. The mixture was filtered, and the filtrate was subjected to centrifugation to remove bulk plant material. The supernatant was collected and clarified by adding 1% Triton X‐100. After centrifugation the supernatant was collected and processed by adding 4% PEG 8K and 0.2 m NaCl to precipitate virus. The pellet was centrifuged, resuspended in buffer, and purified by sucrose gradient.

*Fabrication of Virus Based Scaffolds*: 1 mg mL^−1^ TMV, TYMV, CPMV, 10 mg mL^−1^ TVCV, and 2.67 mg mL^−1^ PVX in aqueous solution 0.7 mL were dropped into 12‐well plates that were coated with poly‐*d*‐lysine using protocol suggested by Corning. The virus solutions were incubated with the PDL coated plate under sterile cells culture hood for overnight. Then the bottoms of each well were rinsed briefly with 18.2 mΩ water before used for BMSCs culture.

*Surface Characterization of Virus Based Scaffolds by AFM*: The surface morphology of virus based scaffolds was observed by AFM (Nanoscope IIIA MultiMode AFM (Veeco)). The bottoms of each 12‐well plate were cut out after virus coating and rinsed with 18.2 mΩ water, then dried with a stream of nitrogen gas before mounting onto AFM sample holder for imaging in the tapping mode.

*BMSC Isolation and Expansion*: Primary BMSCs were isolated from the bone marrow of young adult 80 g male Wister rats (Harlan Sprague‐Dawley Inc.). The procedures were performed in accordance with the guideline for animal experimentation by the Institutional Animal Care and Use committee, School of Medicine, University of South Carolina. Cells were maintained in primary media (Dulbecco's modified Eagle's medium (DMEM) supplemented with 10% fetal bovine serum (FBS), penicillin (100 U mL^−1^), streptomycin (100 μg mL^−1^), and amphotericin B (250 ng mL^−1^)), kept at 37 °C in a CO_2_ incubator with 95% air/5% CO_2_ and passaged no more than seven times after isolation. To induce osteogenesis, primary media were replaced with osteogenic media consisting of DMEM supplemented with 10% FBS, penicillin (100 U mL^−1^), streptomycin (100 μg mL^−1^), and amphotericin B (250 ng mL^−1^), 10 × 10^−3^ m sodium β‐glycerolphosphate, l‐ascorbic acid 2‐phosphate (50 μg mL^−1^), and 10^−8^ m dexamethasone. Media were replenished every 3–4 d.

*RT‐qPCR Analysis*: PDL and virus coated substrates were seeded with 4.0 × 10^4^ cells well^−1^ in primary media and allowed to attach overnight. The unseeded cells were used as a control to normalize the change in gene expression. The media were replaced to osteogenic media and cultured for 6 h, 4 d, 7 d, and 14 d. The cell cultures were terminated at these time points and total RNA was subsequently extracted using E.Z.N.A. RNA Isolation Kit, OMEGA. At least two separate experiments were conducted with each type of sample. The purity and quantity of the extracted RNA were analyzed using Thermo Scientific Nanodrop 2000c spectrophotometer and was reverse transcripted by qScript cDNA Supermix (Quanta Biosciences). RT‐qPCR (iQ5 real‐time PCR detection system Bio‐Rad Laboratories) was done by the method described as: 60 cycles of PCR (95 °C for 20 s, 58 °C for 15 s, and 72 °C for 15 s), after initial denaturation step of 5 min at 95 °C, by using 12.5 μL of iQ5 SYBR Green I Supermix, 2 pmol μL^−1^ of each forward and reverse primers and 0.5 μL cDNA templates in a final reaction volume of 25 μL. Glyceraldehyde 3‐phosphate dehydrogenase (GAPDH) was used as the house keeping gene. Data collection was enabled at 72 °C in each cycle and *C*_T_ (threshold cycle) values were calculated using the iQ5 optical system software version 2.1. The expression levels of differentiated genes and undifferentiated genes were calculated using Pfaffl's method (M. W. Pfaffl, G. W. Horgan, and L. Dempfle, Relative expression software tool) for group‐wise comparison and statistical analysis of relative expression results in real‐time PCR, using GAPDH as the reference gene. Quantification of gene expression was based on the *C*_T_ value of each sample which was calculated as the average of at least two replicate measurements for each sample analyzed. “Pairwise fixed reallocation randomization test” was performed on each sample and a value of *p* < 0.05 was regarded as significant. The primers used for RT‐qPCR are shown in Figure S4 (Supporting Information). Three independent experiments were performed and analyzed for each gene expression study.

*ALP Activity*: After 4 and 7 d of induction in the osteogenic media, the BMSCs seeded on PDL and virus coated substrates were determined as number of cells on each substrate by CellTiter Blue assay. Then the cells were fixed with 4% paraformaldehyde for 15 min at room temperature prior to analyze ALP activity by incubating the briefly fixed cells with 1‐Step *p*‐nitrophenylphosphate solution (Thermo Scientific) for 15 min at room temperature. The solution was transferred to a new microfuge tube containing 250 μL of 2 n NaOH and the absorbance at 405 nm was measured. The measured ALP activity from each sample was normalized to the corresponding cell number. Three independent experiments were performed and analyzed for ALP activity.

*Alizarin Red Staining and Quantification*: Calcium deposition on each substrate was visualized and quantified to confirm and compare osteogenic differentiation by Alizarin red staining. Fixed cell on day 7 were stained with 0.1% Alizarin red solution (Sigma‐Aldrich) pH 4.1–4.5 for 30 min in the dark. The samples were washed with water (18.2 MΩ) prior to imaging. To quantify the amount of dye on each substrate, 300 μL of 0.1 n NaOH was added to each sample to extract the dye from the sample. The extracted dye solution measured the absorbance at 548 nm wavelength. The measured absorbance from each sample was normalized to the corresponding cell number from CellTiter Blue assay. Three independent experiments were performed and analyzed for Alizarin red staining and quantification.

*Immunofluorescence Assays and Image Analysis*: For immuno­fluorescence assays and image analysis, PDL or viral particles coated glass coverslips were used as substrate for BMSCs culture. The substrates were seeded with 4.0 × 10^5^ cells sample^−1^. The cultures were terminated at 24 h after seeding to be used as vinculin immunostaining samples, 6 h after osteoinduction for BMP2 immunostaining analysis and 14 d after osteoinduction for osteocalcin immunostaining study. After termination, cells were fixed in 4% paraformaldehyde at room temperature for 30 min. Each of the samples was then permeabilized for 20 min by 0.1% Triton‐X 100 for 15 min and blocked in 1.5% bovine serum albumin (BSA, Sigma Aldrich) in PBS for 1 h at room temperature. After the blocking, the cells were incubated overnight with mouse monoclonal antibody targeting BMP2 (R&D Systems) at 1:100 dilution in blocking buffer or rabbit polyclonal antibody targeting osteocalcin (Santa Cruz Biotechnology) at 1:100 dilution in blocking buffer or mouse monoclonal antibody targeting vinculin (Neomarkers) at 1:200 dilution in blocking buffer. After overnight incubation, secondary goat antimouse antibody conjugated with fluorescein (Chemicon) was used at 1:400 dilution for 2 h at room temperature with BMP2 and vinculin samples. Secondary goat antirabbit antibody conjugated with Alexa Fluor 546 (Invitrogen) was used at 1:800 dilution for 2 h at room temperature with osteocalcin samples. Rhodamin phalloidin (1:100 in PBS) was used to stain filamentous actin in BMP2 and vinculin samples. Fluorescein phalloidin (1:500 in PBS) was used to stain filamentous actin in osteocalcin samples. Nuclei were stained with DAPI (4,6‐diamidino‐2‐phenylindole, 100 ng mL^−1^). The samples were then mounted and sealed with clear nail polish before imaging. Images of the stained substrates were taken on an Olympus IX81 fluorescent microscope. SlideBook 5 was used to select and analyze immunofluorescence images of vinculin. After setting the threshold for masks, the criteria used to select vinculin spots to be analyzed were XY shape factor larger than 1.5 and area size between 0.5 and 1.5 μm^2^. The average size of vinculin for each image was calculated, followed by the calculation of average vinculin size of cells on PDL and virus substrates and the standard deviation from average values of three individual images which provide more than 500 vinculins for analysis per sample. Immunofluorescence heat maps of actin and vinculin were generated by ImageJ software. Color histogram was generated by measuring pixel intensity across the immunofluorescence heat maps of representative cells on each substrate.

*Spatial Distribution Analysis of BMSCs Cultured on PDL and Virus Coated Substrates*: The spatial distribution of the cells on different substrates was analyzed by NIH ImageJ and R (http://www.R‐project.org) software packages. The fluorescence images of cell nuclei were primarily processed with ImageJ to be presented as particles, and their centroid coordinates were determined. These data were then imported into R for nearest‐neighbor analysis using the SpatStat module. The spatial distribution patterns of cells were identified for 70–90 cells on each substrate.

## Supporting information

As a service to our authors and readers, this journal provides supporting information supplied by the authors. Such materials are peer reviewed and may be re‐organized for online delivery, but are not copy‐edited or typeset. Technical support issues arising from supporting information (other than missing files) should be addressed to the authors.

Supplementary
