# Supplementary material for: Virus Nanoparticles Mediated Osteogenic Differentiation of Bone Derived Mesenchymal Stem Cells
Source: Adv Sci (Weinh). 2015 Jun 25;2(10):1500026. doi: 10.1002/advs.201500026 (PMC5115314; doi:10.1002/advs.201500026)
Supplement: Supplementary file 1 — Supplementary [file ADVS-2-0q-s001.pdf]

Copyright WILEY-VCH Verlag GmbH & Co. KGaA, 69469 Weinheim, Germany, 2015.

ADVANCED SCIENCE

Open Access

Supporting Information

for *Adv. Sci.*, DOI: 10.1002/advs.201500026

Virus Nanoparticles Mediated Osteogenic Differentiation of Bone Derived Mesenchymal Stem Cells

*Kamolrat Metavarayuth, Pongkwan Sitasuwan, Jittima Amie Luckanagul, Sheng Feng, and Qian Wang**

Supporting Information

Virus nanoparticles mediated osteogenic differentiation of bone derived mesenchymal stem cells

Kamolrat Metavarayuth, Pongkwan Sitasuwan, Jittima Amie Luckanagul, Sheng Feng, Qian Wang*

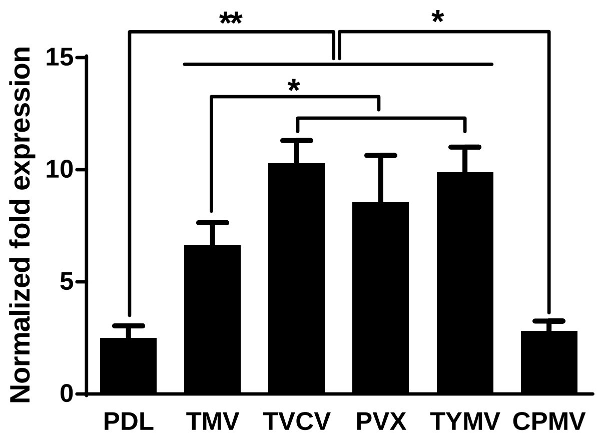

Figure S1. RT-qPCR analysis showed significant BMP2 upregulation in cells grown on TMV, TVCV, PVX, and TYMV coated substrates.

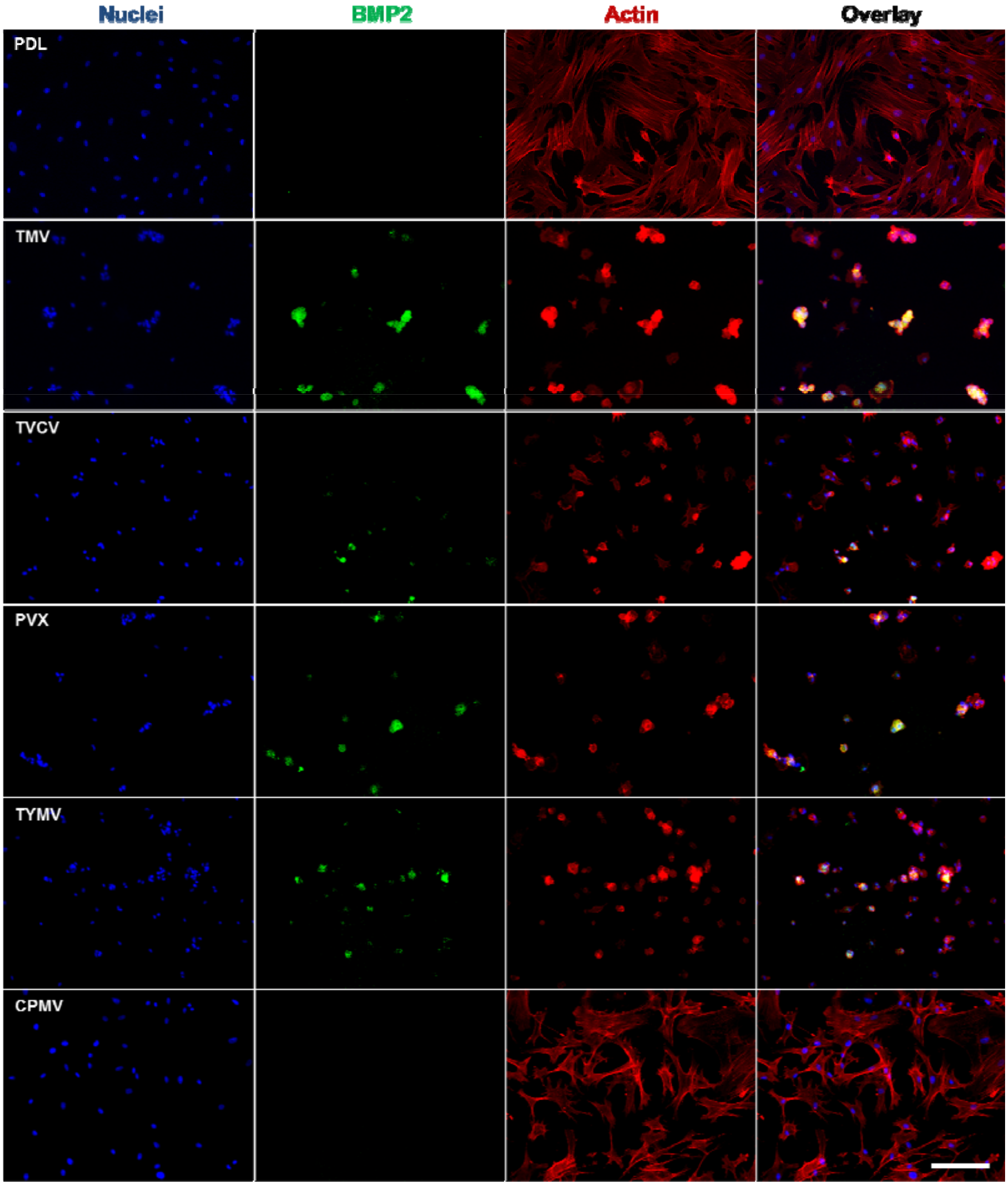

Figure S2. BMP2 immunohistochemical staining suggests the protein expressions are localized to the cell aggregates; most are found on TMV, TVCV, PVX, and TYMV substrates. Scale bar is 200 µm. Color representation: nucleus (blue), BMP2 (green), actin (red).

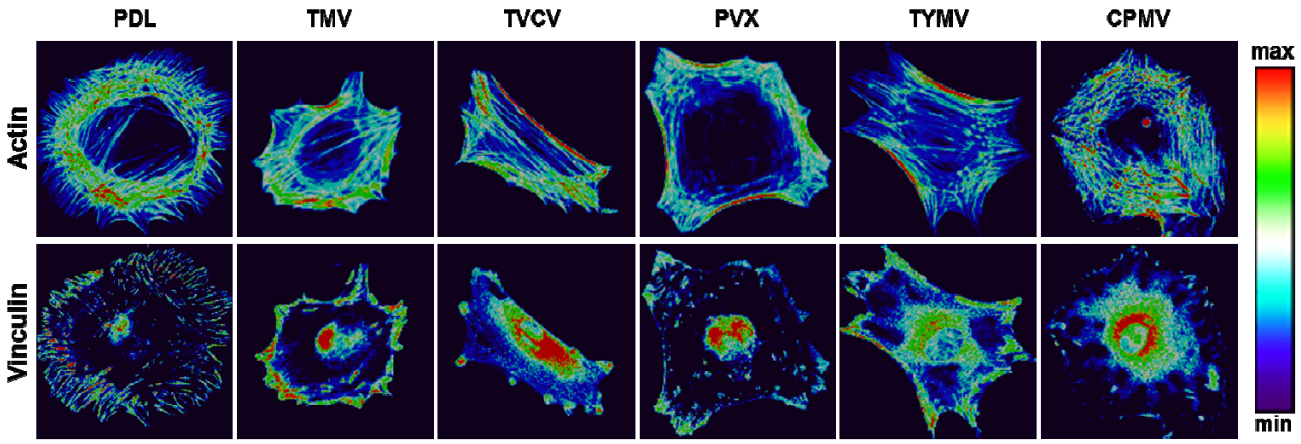

Figure S3. Representative actin (top panel) and vinculin (bottom panel) immunofluorescent heat maps of cells culture on PDL and virus coated substrates.

Table S1. Primers used for RT-qPCR to measure gene expression levels. BGLAP: bone-gamma-carboxyglutamate protein; BMP2: bone morphogenetic protein 2; SPP1: secreted phosphoprotein. 1

Gene	Sequence (5'-3')
BGLAP	Forward: AAAGCCCAGCGACTCT
	Reverse: CTAAACGGTGGTGCCATAGAT
BMP2	Forward: ACCAACCATGGGTTTGTGGTGGAAGT
	Reverse: TCCGCTGTTTGTGTTTGGCTTGACG
GAPDH	Forward: ACTAAAGGGCATCCTGGGCTACACTGA
	Reverse: TGGGTGGTCCAGGGTTTCTTACTCCTT
SPP1	Forward: GACGGCCGAGGTGATAGCTT
	Reverse: CATGGCTGGTCTTCCCGTTGC
